# Early Rehabilitation after Surgical Repair of Medial and Lateral Collateral Elbow Ligaments: A Report of Three Cases

**DOI:** 10.3390/ijerph17176133

**Published:** 2020-08-24

**Authors:** Seong Eon Kim, Yong Chul Choi, Ji Young Lee

**Affiliations:** 1 Department of Physical Education, Sejong University, Seoul 05006, Korea; kimpulse@naver.com; 2 Department of Physical Education, Gangneung-Wonju National University, Gangneung-si 25457, Korea

**Keywords:** elbow, injury, ligament, rehabilitation, surgery

## Abstract

Elbow ligament injuries are commonly caused by overuse; degeneration; and trauma; such as from a fall or collision. The purpose of this study was to present the results of three cases involving patients undergoing early rehabilitation after surgical treatment for complex injury of the elbow medial collateral ligament (MCL) and lateral collateral ligament (LCL). Two patients were non-athlete middle-aged women and one was a recreational judo player. Surgery was performed through open incision or arthroscopically. Rehabilitation consisted of range of motion (ROM) exercise; muscle strength restoration; and neuromuscular training. Passive ROM exercise and isometric strength exercise began at 7 days; isotonic strength training at 6 weeks; and neuromuscular training at 3 months after operation. Center- and home-based methods of exercise participation were combined. Center-based exercises were performed 1–2 times per week for the first 6 months and 1–2 times per month for the next 6 months. Patients also performed home-based and self-monitoring exercise. Examinations included ROM using a goniometer; muscle strength test using isokinetic equipment; and Oxford elbow score. In the six months after surgery; flexion ROM was 130° for Case A (health side 145°), 110° for Case B (health side 145°), and 135° for Case C (health side 135°); grip strength was restored to 13 kg (health side 28 kg), 16 kg (health side 25 kg), and 38 kg (health side 52 kg); and isokinetic flexion strength was improved to 30 Nm (health side 58 Nm), 21 Nm (health side 50 Nm), and 72 Nm (health side 80 Nm), respectively. In conclusion; patients who underwent early rehabilitation recovered ROM and muscle strength and returned to daily activity without any side effects. This study showed that patients with elbow MCL and LCL injuries took approximately 3 months to recover meaningful ROM; approximately 6 months to recover muscle strength; and 4–8 months to play light recreational sports. In addition; it took patients 6 weeks to return to their daily activities and 6 months to improve questionnaire scores in their function and pain during daily activity. In follow-up two years after surgery; all three patients had full ROM and muscle strength within 10% of the healthy side

## 1. Introduction

Elbow ligaments increase joint stability when muscles exert tension or facilitate elbow movements, such as flexion, extension, pronation, and supination [1]. Elbow ligament injuries are caused by degeneration, repetitive overuse, and traumatic injuries such as falls [2]. If ligament injury occurs, surgical or non-surgical treatment is often advised, depending on instability status [3]. In principle, non-surgical treatment is preferred as the primary treatment, but surgery should be performed if persistent pain, loss of function, and joint instability are present [4]. It has been previously reported, however, that only 42% of patients had a successful return after non-surgical treatment for elbow ligament injury [5]. In contrast, an investigation of athletes’ return to sports after injury and operative treatment found that 83% of participants returned to sports within 1 year after surgery, while the remaining 17% failed to return to their previous activity levels during this time period [6].

Rehabilitation in a patient with surgical treatment remains a formative factor in the patient’s successful return after injury because it improves the muscle strength and functionality required for daily life and competition and increases joint stability [7]. A study of elbow medial collateral ligament (MCL) and lateral collateral ligament (LCL) injuries caused by dislocation indicated that daily functionality became possible at 6 weeks after surgery and light sports at 3 months after surgery [8]. In addition, in a study in which throwing began after surgery, rehabilitation took, on average, 4.4 months and patients were, on average, able to return to games after 11.6 months [6]. Another study involving a large number of ulnar collateral ligament patients in which range of motion (ROM) and concentric strength exercises were started 2 and 3 weeks, respectively, after surgery reported that it took 5 months to return to the game after surgery and rehabilitation [9].

As the frequency of injury to the elbow MCL is relatively high in athletes in sports that require repetitive overhead motions such as throwing, there are many related rehabilitation studies [9,10]. In addition, one study introduced a rehabilitation program conducted immediately after surgery [10]. However, LCL single injury or MCL and LCL complex injury is often caused by trauma, such as dislocation, and the frequency of damage and related studies are less than for MCL [11,12]. Although some studies have indicated that rehabilitation is possible immediately after surgery, the results of studies on cases are very rare [13,14]. Therefore, although the present study involved only three patient cases, the patients were non-professional athletes with elbow MCL and LCL complex injuries. In addition, the rehabilitation program performed early ROM exercise on the 7th day after surgery, started isometric muscle training, and attempted to report the results to the 2-year follow-up. The researchers hypothesized that the application of early rehabilitation would not be dangerous and would effectively restore ROM and muscle strength to return to daily life.

## 2. Materials and Methods

### 2.1. Patients

Three patients underwent surgery by an orthopedic surgeon. After consultation, the specialists referred patients who agreed to participate in rehabilitation to the sports medicine center. Patients referred to a rehabilitation center were assured of safety regarding surgical wounds and X-rays and were provided a self-symptom consultation by a specialist. During the initial 6 months, patients visited the center 1–2 times a week to exercise; over the next six months, they visited the center 1–2 times a month to learn how to exercise and practice at-home exercises. One year after completion of a 1-year rehabilitation session, patients revisited the center for examination and consultation. The researcher recorded the test results and rehabilitation processes and explained that the contents could be used for research purposes. All patients submitted written informed consent. The study was approved by the researcher’s institutional review board center (approved number: GWNU IRB 2020-16) and conducted in accordance with the Helsinki Declaration. Patient information is summarized in Table 1.

**Figure 1:**
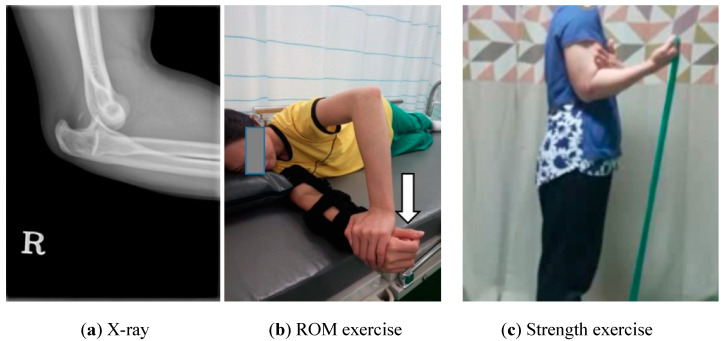
Case A’s X-ray, and range of motion (ROM) and strength exercise.

Patient A: Patient A was a 39-year-old housewife who swam recreationally five days a week. She fell while crossing the crosswalk and her right arm was twisted and injured as her hand hit the ground. Following the injury, she immediately went to the emergency room by ambulance. An X-ray examination showed complete dislocation of the right elbow without bone fracture (Figure 1a). After relocation of the joint, her elbow was immobilized with a cast and the patient returned home. Four days later, upon moving her hand and wrist, the patient’s elbow re-dislocated and she returned to the emergency room. She underwent magnetic resonance imaging (MRI) and was diagnosed with a MCL partial tear, LCL complete tear, and common extensor tendon complete tear. The surgeon decided to operate only on the LCL using the open method and a primary ligament repair was conducted by connecting a wire to the distal humerus hole (Table 1).

Patient B: Patient B was a 60-year-old woman whose profession was in the field of agriculture. She played badminton recreationally 1–2 days a week. She had no history of elbow trauma, but had suffered elbow pain for 2 years. During this period, she intermittently attended physical therapy and took pain relievers, but did not improve. Recently, she experienced elbow tingling and nighttime pain and accordingly visited the hospital. MRI examination revealed significantly progressed degenerative transformations of the MCL and LCL. Due to the patient’s advanced age, the specialist recommended rest and conservative treatment rather than immediate surgery. However, despite six months of conservative treatment, the patient’s pain intensified; in response, the specialist decided to operate using arthroscopy. She underwent medial and lateral ligament repair and debridement of the surrounding tissues by arthroscopic primary suture (Table 1).

Patient C: Patient C was a 17-year-old male high school student and judo recreational player. During a judo match, his elbows were severely strained due to excessive twisting and hyperextension without complete dislocation. The patient immediately experienced edema and severe pain. After visiting the local hospital and completing an X-ray, the patient’s elbow was immobilized with a cast for 3 weeks. Thereafter, the patient continued to receive physical therapy for 2 months; however, the pain persisted and muscle strength was greatly diminished. To attain a more precise and accurate diagnosis, the patient was admitted to a general hospital where significant tears were observed in the MCL and LCL. Considering the patient’s age and activity level as well as the fact that he had little functional strength 2 months after injury, the specialist decided to perform arthroscopic surgery and reconstructed the MCL using a palmaris longus auto-graft (Table 1).

### 2.2. Rehabilitation Procedure

The rehabilitation program referred to previous studies on elbow, shoulder, and upper body rehabilitation [7,9,10,15]. Each phase of rehabilitation exercises are summarized in Table 2. Rehabilitative treatments, including ROM exercises, strength training, and neuromuscular training, were performed according to general guidelines [10]. A long arm brace was applied for 6 weeks after operation and tolerable passive ROM (PROM) exercises were initiated at 7 days after surgery (Figure 1b). Light strength exercises with elastic bands were started at 6 weeks after surgery (Figure 1c) and neuromuscular training with various equipment was started at 3 months after surgery. Patients performed center-based and home-based exercises together. Up to 1 year after surgery, they made regular visits to the sports medicine center, after which they only visited intermittently. All patients completed a 2-year follow-up.

ROM Exercise and Mobilization: PROM flexion and extension were implemented at 7 days after surgery to prevent joint stiffness and pronation and supination were started after 3 weeks after surgery with tolerated pain (Figure 1b). Six repetitions of static stretching for 10 s were performed at least six times a day. Self-massage and hot-packs were also advised for pain management and tissue relaxation.

Strength Exercise: The early phase strength exercise was an isometric contraction exercise that was performed from time to time with a brace and added hand grip. As isotonic exercise, elbow flexion, and extension were also performed using elastic bands (Therabands^©^, Hygenic Corp., Akron, OH, USA) from 6 weeks after surgery (Figure 1c). Bands were used gradually, increasing the intensity from weakest to strongest, and patients were instructed to perform this exercise twice a day. After 3 months, advanced strength training was performed using weight machines and dumbbells such as the chest press and arm curl.

**Figure 2:**
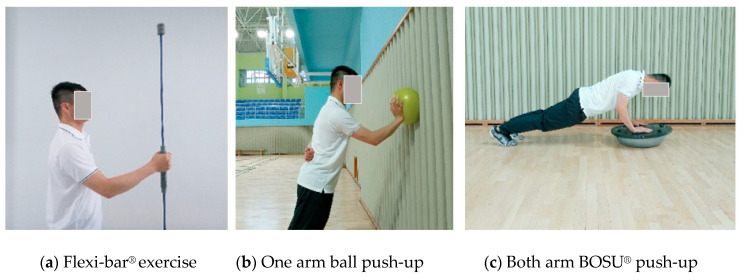
Neuromuscular training.

Neuromuscular Training: Neuromuscular training began at 3 months. Neuromuscular training included push-ups with BOSU^®^ (NexGen™, Ashland, OH, USA), a gym ball (Therabands^©^, Hygenic Corp., Akron, OH, USA), and vibration exercise with Flexi-bar^®^ (Flexi-bar Inc., München, Germany). The initial exercise began with standing wall push-ups using the gym ball, followed by kneeling push-ups with the gym ball. Exercise intensity was increased from weak to strong using a ground stable BOSU^®^, a large gym ball, and the smallest, most unstable ball (Figure 2).

### 2.3. Measurement: ROM, Strength, and Oxford Elbow Score

ROM measured the total range from extension to flexion with the goniometer. Reference ROM degree, based on the healthy elbow, was 145° for Cases A and B and 135° for Case C.

The isokinetic dynamometer test (HUMAC, Lumex Inc., Ronkomma, NY, USA) was performed at 30°/s. Measurements were taken at 3 months for safety. Inspection procedures were performed according to standard guidelines [16]. Elbow flexion and extension strength were measured in a supine position and the maximum value occurred after four repetitions.

Grip strength was measured using a grip dynamometer (Takie5401, Japan). Standing in a wide-shoulder posture with feet wide open, the arm was lowered. The machine was aligned with the second metacarpal fingers twice before the maximum strength was measured.

The elbow questionnaire was based on the Oxford Elbow Score (OES), which uses a range from 0 to 4 and includes 12 items. The overall score range is 0–48, with 0 as the “worst” elbow condition and 48 meaning the “normal” elbow. The OES asks questions related to difficulties in daily activities such as washing, carrying shopping bags, and dressing due to elbow problems in the past 4 weeks and pain or discomfort in various conditions [17].

## 3. Results

**Figure 3:**
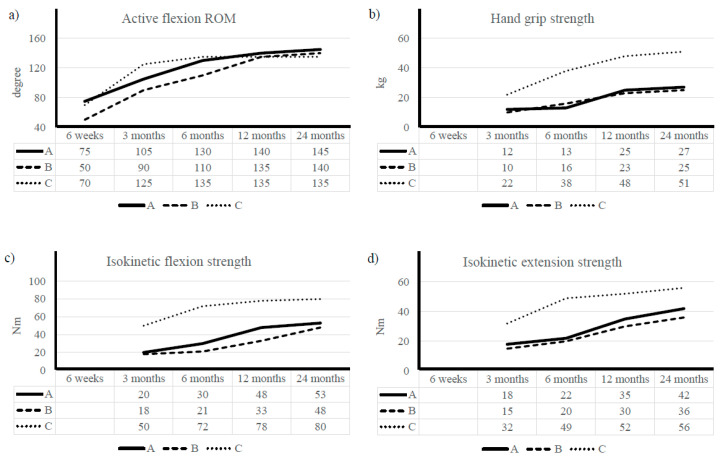
Results of ROM and strength. ROM, range of motion; Nm, newton meter. (**a**) Healthy side elbow flexion ROM: Case A; 145°, Case B; 145°, Case C; 135°. (**b**) Health side hand grip strength: Case A; 28 kg, Case B; 25 kg, Case C; 52 kg. (**c**,**d**) Healthy side isokinetic strength of Flexion and Extension: Case A; 58 and 46 Nm, Case B; 50 and 39 Nm, Case C; 80 and 57 Nm.

After 2 years of follow-up, a normal X-ray was observed for all patients and physical examination showed no instability and significant discomfort. The results for ROM, muscle strength, and OES over time are shown in Figure 3 and Table 3.

Case A: Flexion-extension ROM was 75° at 6 weeks, 105° at 3 months, and almost recovered at 6 months. In isokinetic strength, initial flexion was 20 Nm and extension 18 Nm. At 1 year, flexion reached 48 Nm and extension 35 Nm; by two years, the patient demonstrated 90% recovery in these elements. Grip strength steadily increased from 12 to 27 kg. She returned to swimming at 6 months. The OES was 12 at 3 months and 30 at 12 months.

Case B: Flexion-extension ROM was 50° at 6 weeks, 90° at 3 months, and almost recovered at 6 months. Flexion and extension isokinetic strength were 18 Nm and 15 Nm at 3 months, 33 Nm and 30 Nm at 1 year, and 48 Nm and 36 Nm at 2 years, respectively. Grip strength increased from 10 to 25 kg. She started carefully playing badminton at 8 months. The OES was 9 at 3 months and 34 at 12 months.

Case C: Flexion-extension ROM was 70° at 6 weeks and almost recovered at 3 months. Flexion and extension isokinetic strength were 50 Nm and 32 Nm at 3 months, 78 Nm and 52 Nm at 1 year, and 80 Nm and 56 Nm at 2 years, respectively. Grip strength increased from 22 to 51 kg. He started to slowly return to practicing judo at 4 months. The OES was 22 at 3 months and 42 at 12 months.

## 4. Discussions

This study presented three patients participating in rehabilitation after ligament surgery; the participants were followed for 2 years to survey their recoveries from chronic and traumatic elbow injuries. The main findings of this study were that the participants had mostly recovered ROM at 3 months and strength after 6 months. At 2 years, the ROM demonstrated an approximately 10% deficit.

Because there are limited studies related to elbow ligament complex injuries, rehabilitation was carried out according to general physiological reflexes and guidelines. Physiologically, the healing of skin sutures typically takes 2–3 weeks and the tendon and muscle suture area typically remains stable during tension for 6 weeks [10,18]. Several prior studies recommend starting ROM exercises as early as possible after surgery [19,20]. A study of rotator cuff patients compared an early group performing ROM exercise 1 day after arthroscopic surgery and a delay group performing ROM exercise after immobilization for 6 weeks. The results showed that the early group demonstrated lower pain and faster ROM improvements [21,22]. The reason for emphasizing early ROM is stiffness. Stiffness, which occurs after injury or after surgery, occurs in at least 5% of patients, causes pain, and can impair functional recovery after surgery [23]. Our study, which started 7 days after surgery, considered patient fears, pain, and stability in the wound area. In addition, we followed the guidelines recommending absolute immobilization for 6 days to protect the wound [10]. General guideline suggested post operation exercise such as elbow isometric strength training, handgrip motions, and scapular mobilization [10]. Additionally, the guideline for throwing athletes recommended full ROM at 4–6 weeks after surgery; however, in this study, it took approximately 3–6 months to regain ROM. Notably, it was difficult to advise aggressive ROM exercise for non-athletes. Patients may have been slow to recovery ROM due to complex medial and lateral injuries.

Existing literature recommends starting strength training exercises at 6–10 weeks after surgery [10,24]. Taking this suggestion into account, in this study, strength training began at 6 weeks and was conducted with flexion and extension. In addition, the pronation and supination strength training started at 3 months. This is because stress has been found to biomechanically impact ligament instability in the valgus and varus positions [11]. Physiologically, representative mechanisms of muscle strength improvement appear due to neuromuscular adaptation and hypertrophy [25]. In general, it has been reported that 12 weeks of muscle hypertrophy was required as a result of resistance training in healthy people [26]. And although it was a lower extremity study, the 6-week muscle training effect was positive [27,28]. As a result of this study, muscle strength recovery took about 3–6 months for young men, but was relatively slow in women. These causes cannot be excluded from differences according to pre-injury muscle strength and rehabilitation participation rate.

A previous study that documented recovery after multiple MCL and LCL injuries stated that daily functioning returned after 6 weeks and light sports activities could be performed after 3 months [8]. Similarly, patients in this study took 6 weeks to return to their daily activities. However, since the OES is 4–10, it would have been a big inconvenience to everyday life. In one previous study, the lowest OES of asymptomatic people in their 40s and 50s was a score of 12 or 16 [17]. The 6-month OES of this study was 22 in Cases A and B and 30 in Case C. Although our patients took 6 weeks to return to their daily activities, they took approximately 4–8 months to return to recreational activity. This delayed return to sports may be due to the fact that the exercises in which the patients participated—swimming, badminton, and judo—involved frequent arm use and muscle strength recovery delayed. The diagnostic criteria for muscle sarcopenia suggest that the minimum grip strength necessary for daily life is 18 kg for women and 26 kg for men [29]. The patients in this study took approximately 6 months to restore their strength to this level.

The rehabilitation process in this study has several characteristics. First, it includes ROM exercise, which for the safety of the wound, is recommended to begin at 2 weeks after surgery [9]; however, in other programs, it is recommended to be performed immediately after surgery to reduce stiffness, pain, and inflammation [10]. Based on the results from ROM with other joints [19,30], this study was based on the premise that early ROM exercise is safe and has many advantages. Considering the fact that the MCL and LCL were injured in the patients in this study, ROM exercise was delayed until the 7th day after surgery, which is still relatively early. In addition, the supination and pronation exercises were performed carefully at 3 weeks since valgus and varus stress is dangerous in patients with this complex injury [11].

Another component of our rehabilitation program is elbow isotonic strength training, which is generally recommended at 6 weeks after surgery [9,10]. However, it was said that the scapular mobilization exercise, grip exercise, and isometric elbow muscle exercise, which does not negatively affect the surgical site, can be performed immediately after surgery [10]. Therefore, in this study, strength training was actively performed from the beginning and elbow flexion and extension isotonic strength training was performed at 6 weeks.

The final stage of our rehabilitation program is neuromuscular training. Neuromuscular training serves to increase joint stability and performance in dynamic sports and decrease the risk of re-injury [31]. In general, neuromuscular training includes proprioceptive exercise and plyometric, functional, and balance training, using an unstable tool or the position-reposition method [24]. Previous studies suggested that strength training was to be performed at 6 weeks and plyometric training at 6 or 10 weeks [9,10]. Considering that the patients in the present study are non-professional athletes, neuromuscular training began at 3 months and patients completed it in a standing position for safety.

Although only including three case reports, the present study offers post-operative long-term rehabilitation information about rare patients suffering from elbow MCL and LCL complex injuries. In our study, ROM and strength training were performed early, but neuromuscular training progressed slowly. As a result, patients returned to their daily life by restoring muscle strength, function, and X-ray finding. To determine whether these results are effective over time, further studies are warranted.

This study has the following limitations. Due to the small number of patients, it was not possible to clearly uncover differences between recovery from complex and simple ligament injuries; the impact of sex, age, and high muscle strength before injury also could not be assessed. As patient’s sex, age, injury mechanisms, and surgical methods differed, the generalizability of the study’s results are limited. Patients had difficulty in continuing to visit the center due to distance and work, so the proportion of home-based workouts was high. Also, the patient’s exercise frequency was not investigated in this study. In the future, it may be necessary to collect more cases and conduct further research with case-controlled studies on rehabilitation methods.

This study was one of the rare studies that applied early rehabilitation to patients with complex medial and lateral ligament injuries. Although this study is limited to generalization due to the small number of participating patients, it was reported that ROM, isometric strength exercise, and upper limb exercise were safe and positive for pain management and function recovery in patients after ligament surgery.

## 5. Conclusions

Early postoperative rehabilitation of patients with complex medial and lateral elbow injuries restored ROM and muscle strength and returned to daily activities without side effects. They took about 3 months to recover meaningful ROM, about 6 months to restore strength, and 4–8 months to play light recreational sports. In addition, it took the patients 6 weeks to return to their daily activities and improvement in function and pain during daily activities took 6 months in the questionnaire evaluation. At follow-up 2 years after surgery, all patients had complete ROM and muscle strength within 10% of the healthy side.

## Figures and Tables

**Table 1:** Summary of patients.

	A	B	C
Sex/age	Women/39	Women/60	Men/17
Job	Housewife	Farmer	Student
Recreational sports	Swimming	Badminton	Judo
Cause and Mechanism	Trauma, Dislocation	Chronic, Degeneration	Trauma, Twist and hyper-extension
Surgery method	Open	Arthroscopy	Arthroscopy
Restoration of daily life	6 weeks	6 weeks	6 weeks
Return to recreational activity	6 months	8 months	4 months

**Table 2:** Summary of rehabilitation exercise.

Phase	Time	Exercise
Phase 1	7 days	PROM exercise flexion, extension Isometric strength exercise Hand grip exercise Scapular mobilization
Phase 2	3 weeks	Continue phase 1 PROM exercise supination, pronation
Phase 3	6 weeks	Continue phase 2 Light strength exercise Elbow and wrist: flexion and extension with elastic band
Phase 4	3 months	Continue phase 3 Advanced strength Elbow and wrist: flexion and extension with dumbbell Elbow and wrist: pronation and supination with tube band Machine weight and dumbbell (chest press, pull down, et al.) Neuromuscular training gym ball and small ball push up with standing position
Phase 5	6 months	Continue phase 4 Aggressive strength Emphasize eccentric contraction Neuromuscular training BOSU^®^, gym ball push up with kneel down position Vibration exercise with Flexi-bar^®^ Medicine ball catch and throwing

PROM, passive range of motion.

**Table 3:** Oxford elbow score.

Patients	6 Weeks	3 Months	6 Months	12 Months	24 Months
Case A	7	12	22	30	44
Case B	4	9	22	34	43
Case C	10	22	30	42	46
